# Optimising daily sampling of plant–bee interaction networks

**DOI:** 10.1111/1365-2656.70309

**Published:** 2026-07-15

**Authors:** Caio S. Ballarin, Jorge Isla, Blanca Arroyo‐Correa, Elena Quintero, Leandro Hachuy‐Filho, Felipe W. Amorim, Pedro Jordano

**Affiliations:** ^1^ Laboratório de Ecologia da Polinização e Interações—LEPI, Departamento de Biodiversidade e Bioestatística, Instituto de Biociências Universidade Estadual Paulista “Júlio de Mesquita Filho” (UNESP) Botucatu São Paulo Brazil; ^2^ Programa de Pós‐graduação Em Biologia Vegetal, Instituto de Biociências Universidade Estadual Paulista “Júlio de Mesquita Filho” Botucatu São Paulo Brazil; ^3^ Integrative Ecology Group Estación Biológica de Doñana, EBD‐CSIC Sevilla Spain; ^4^ Departamento de Biología Vegetal y Ecología Universidad de Sevilla Sevilla Spain; ^5^ Programa de Pós‐graduação Em Zoologia, Instituto de Biociências Universidade Estadual Paulista “Júlio de Mesquita Filho” Botucatu São Paulo Brazil

**Keywords:** diurnal network structure, dynamical network topology, neotropical savanna, resource utilisation patterns, sampling schedules

## Abstract

1. Plant–pollinator interactions are dynamic not only across seasons but also over hourly time scales, even beyond the day–night contrast. However, capturing the daily temporal variability of pollination networks during field data collection is challenging. Consequently, our understanding of daily variation in network composition and structure remains incomplete due to limited sampling coverage. Moreover, inaccurate sampling protocols can result in missing links that impair the identification of underlying processes and emerging patterns. We address this gap by studying the fine‐scale temporal organisation of the diverse diurnal plant–bee relationships and examining how the interaction composition and network structure fluctuate throughout the day.
2. We extensively sampled plant–bee interactions in a Brazilian Cerrado over two flowering seasons, encompassing six 2‐h sampling periods throughout the day. We compared interaction β‐diversity and network structural properties across sampling periods. A factorial approach was used to merge interactions among all combinations of sampling periods to assess how the composition and structure of the partial‐day networks deviated from those exhibited by the full‐day network (i.e. consolidating all interactions).
3. Plant–bee interactions exhibited notable variations in network metrics across daily sampling periods. From dawn to dusk, plant–bee networks showed reduced modularity and specialisation but greater nestedness. Interaction β‐diversity between sampling periods increased progressively with wider temporal intervals, mainly explained by interaction rewiring among shared species. Combining four 2‐h sampling periods recovered up to ~80% of all pairwise interactions and ~ 82% of interaction frequencies, and generated partial‐day networks that most closely resembled the structure of full‐day networks. These best‐performing combinations consistently drew from daily central hours, whereas early‐morning and late‐afternoon periods contributed fewer but unique interactions.
4. Our study demonstrates that plant–bee interaction networks are time‐sensitive at the intraday scale and that interaction completeness is not necessarily improved by increasing effort within a single time window, but rather by distributing sampling across, or focusing on, the most informative periods of the day. We provide a general framework to evaluate how temporal sampling design affects network representativeness in systems where interactions depend on time‐of‐day.

## Graphical abstract

Daily variation in ecological interaction networks is rarely quantified due to limited temporal sampling, yet network structure may change substantially within a single day. We show that plant–bee networks exhibit strong intraday shifts in composition and structure, and that sampling distributed across key daily periods better captures network organisation than increasing effort within a single time window. We further provide a general framework to evaluate how temporal sampling design affects network representativeness.

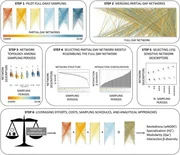

## 1 INTRODUCTION

Virtually all ecological interactions are undersampled (Jordano, 2016; Vázquez et al., 2009), reflecting the inherent limitations of biodiversity sampling (Gotelli & Colwell, 2011; Magurran, 1988) and motivating the need to identify the sources of such undersampling and its potential biases. One reason for incomplete interaction inventories is that interactions are structured in space and time, reflecting circadian, phenological, and ontogenetic cycles, as well as spatial constraints on dispersal that confine interactions to specific spatiotemporal contexts. For instance, a pairwise interaction may occur only in specific habitats (e.g. canopy vs. understorey, or forest interior vs. edge) or time periods (e.g. summer vs. winter or morning vs. afternoon) and be absent otherwise (Jordano, 2016; Olesen et al., 2011).

When ecological networks are treated as static snapshots—by collapsing variation across space and time—interaction patterns and emergent structural properties are assumed fixed, limiting our ability to identify processes shaping interactions, and to predict system responses to environmental change (Poisot et al. 2015; Fründ et al., 2016; Vizentin‐Bugoni et al., 2016). Selecting representative spatiotemporal slices that capture the dynamic behaviour of species interactions is, thus, crucial for studying underlying processes and observed patterns in ecological interaction assemblages, as they may manifest significant variation across spatiotemporal scales and contexts (CaraDonna et al., 2021; CaraDonna & Waser, 2020; Levin, 1992; Olesen et al., 2008, 2010; Schwarz et al., 2020).

A major source of bias underlying discrepancies between realised and potential interactions is the temporal uncoupling among interacting organisms (Jordano, 2016). Contemporary ecology increasingly recognises that ecological interactions vary across temporal scales, and choosing an appropriate temporal resolution is essential to reveal scale‐dependent patterns (Basilio et al., 2006; CaraDonna & Waser, 2020; Schwarz et al., 2020). For example, ecological networks reveal different dynamics across temporal scales, from interannual turnover driven by species migration and extinction at yearly scales (Alarcón et al., 2008; Resasco et al., 2021), to seasonal shifts driven by phenology within years (Arroyo‐Correa et al., 2020; Basilio et al., 2006; Bramon Mora et al., 2020; CaraDonna et al., 2017), resource‐use flexibility and rewiring across months (CaraDonna & Waser, 2020; Olesen et al., 2008) and circadian interaction patterns within days (Baldock et al., 2011; Nagano, 2023; Ratoni et al., 2025). Although less studied, daily variation in interaction patterns is important for understanding within‐day ecological dynamics, offering fine‐scale insights into interaction patterns (Herrera, 2024), particularly in systems involving ectothermic insects, where activity and foraging respond to temperature and rapid changes in resource availability.

Plant–pollinator interaction networks exhibit highly dynamic topologies at daily temporal scales (i.e. Baldock et al., 2011; Nagano, 2023; Ratoni et al., 2025). Intraday changes in interaction networks are expected to emerge from the joint effects of (i) daily rhythms in floral display and reward production (Ballarin et al., 2022), (ii) interspecific differences in pollinator circadian activity and microclimatic sensitivity (Herrera, 2024; Polatto et al., 2014; Stone et al., 1988) and (iii) shifts in the competitive environment as the identity and density of foragers and available resources change throughout the day (Ballarin et al., 2022). These processes imply that different periods of the day represent distinct ecological states of the same community, rather than incomplete samples of a single static network. Consequently, assuming temporal invariance overlooks shifts in interaction strength and resource partitioning, masking how network structure emerges through interaction rewiring and turnover. Static descriptions may therefore misrepresent network structure, attributing differences between networks to environmental drivers when they instead reflect differences in temporal sampling. For example, a pollination network may appear highly specialised if sampling captures periods of strong niche partitioning and floral constancy, even though interactions shift and competition relaxes throughout the day. Even a single missed interaction may distort estimates of, for example, specialisation and disturbance propagation within mutualistic networks.

However, surveys of plant–pollinator interactions usually sample only a subset of daily hours or ignore the timing of each interaction (see Baldock et al., 2011). Although the importance of daily timing is widely acknowledged (Baldock et al., 2011; Nagano, 2023; Ratoni et al., 2025), we still lack a clear understanding of how pollination network structure and interaction composition change across the day and how this affects sampling reliability. Plant–bee interactions are particularly suitable for this purpose because intraday shifts in bee activity and flexible resource use promote frequent interaction turnover and rewiring. Here, using a high‐resolution plant–bee interaction network sampled at six 2‐h intervals (1440 h in total), we (i) quantified how network structure and the identity of pairwise interactions differ among daily sampling periods. To propose field strategies that minimise daily sampling efforts while ensuring interaction data completeness, we then (ii) evaluated all possible combinations of 2‐h intervals to identify sampling schedules that maximise interaction completeness and best recover the full‐day network, used as a benchmark. Finally, we assessed which network metrics are least sensitive to incomplete daily sampling and therefore better preserve information about network structure under reduced sampling effort.

Based on this framework, we hypothesised that both network structure and interaction composition should change predictably across the day, primarily through extensive interaction rewiring rather than species turnover. This expectation follows from the fact that most plants and bees co‐occur throughout the day but adjust their foraging and visitation patterns in response to intraday changes in floral resource presentation and microclimatic conditions, leading the same species to interact with different partners at different times (Arroyo‐Correa, 2025; Ratoni et al., 2025). We further hypothesised that sampling schedules including the central daylight hours, when temperature and bee activity are highest, floral overlap is maximal, and resources are not yet strongly depleted, should maximise interaction completeness and best approximate full‐day network structure. Finally, we expected that network metrics based on interaction frequencies would be less sensitive to incomplete daily sampling, as observed for increasing temporal sampling effort in other contexts (e.g. Vizentin‐Bugoni et al., 2016).

## 2 MATERIALS AND METHODS

### 2.1 Study site and data collection

This study was carried out in a Cerrado area situated in the Botucatu municipality, São Paulo state, Brazil (34 ha; 22°56′20″ S, 48°27′29″ W; 860 m a.s.l.), characterised by the predominance of herbaceous/grassy vegetation interspersed with scattered trees and shrubs. The plant community comprises functionally diverse species with contrasting phenologies, daily flower‐opening schedules, resource types, shapes and colours, with most species being visited by bees even when these are not their primary pollinators (see Ballarin et al. 2025; Ballarin, Amorim, et al. 2026; Ballarin, Fidelis, et al., 2026). To sample empirical plant–bee interactions, we conducted weekly visual censuses over two consecutive flowering seasons (2021/2022–2022/2023) across 12 100 m^2^ (10 × 10 m) plots spaced at least 30 m apart, during the peak of angiosperm flowering (mid‐July to early March). Each census consisted of recording interaction events in six 2‐h intervals, divided over two consecutive days, based on local clock time: from 06:00 to 12:00 on the first day and from 12:00 to 18:00 on the second day. Sampling days were selected to ensure similar weather conditions (e.g. temperature, cloud cover and wind), and the 2‐h intervals were adopted to allow all plots to be surveyed within each period and to minimise the influence of seasonal variation in sunrise and sunset on the earliest and latest intervals. During each observation period, we sampled all bee‐pollinated flowering plant species within each plot (ca. 90% of all animal‐pollinated species; Ballarin, Fidelis, et al., 2026) using a plant‐focused protocol. Groups of nearby flowering individuals were observed for 5 min, and we recorded the number of legitimate bee visits (i.e. visits contacting reproductive structures; *n* = 720 h per flowering season). Our sampling encompassed all bee taxa visiting flowers in plots, including taxa foraging on distinct resources, and species with both temporally restricted (e.g. *Ptiloglossa*) and broad (e.g. *Apis mellifera*) daily activity patterns, reflecting a dataset sensitive to sampling time (Table S1). Bee specimens not reliably identified in the field were collected for later taxonomic identification after floral visits. Collection and identification of bee specimens were conducted under SISBIO/ICMBio authorisation number 75978‐1, and fieldwork was authorised by the municipal authority responsible for the study area. No animal ethics committee approval was required for the invertebrate sampling involved in this study. To mitigate potential biases associated with sampling periods, we varied the order of plot surveys among sampling days.

### 2.2 Composition and structure of empirical networks across sampling periods within the day

We created matrices *M*_*t*_, where elements *m*_*ijt*_ represent the frequency of legitimate interactions between a particular plant species *i* and a specific bee species *j* at daily time interval *t*. To assess variation in network structure according to the daily period of data sampling, we constructed 144 interaction networks, with each network including the aggregated interaction frequencies between plants and bees in a given 2‐h sampling period/plot/flowering season (*n* = 24 plant–bee networks—*M*_*t*_—for each 2‐h sampling period). Thus, each 2‐h period was sampled in 12 plots per season across two seasons, totalling 240 h per period. Interaction richness estimates for each sampling period indicated a coverage of 72.33 ± 3.27% (mean ± SD; Figure S1) of all potential pairwise plant–bee interactions (Chao 1 estimator, ‘iNEXT’ package—version 3.01; in R—version 4.4.2; Chao, 1984; Chacoff et al., 2012; Hsieh et al., 2016). Sampling completeness varied among networks built for each period and plot, with the 16:00–18:00 period consistently showing lower completeness (*χ* ^2^_139,5_ = 22.76; *R* ^2^‐_adjusted_ = 0.70; *p* < 0.01; Figure S1).

We then evaluated whether patterns of plant–bee interactions differ across 2‐h sampling periods using different network descriptors. Pairwise interaction richness was calculated as the number of unique pairwise plant–bee interactions. Connectance (*C*) computes the proportion of realised out of all potential interactions. Interaction evenness (*IE*) assesses the uniformity of interaction distribution. Specialisation degree (*H2*′) measures the degree of specialisation by considering partner availability and interaction exclusiveness (Blüthgen et al., 2006). Weighted nestedness (*wNODF*) evaluates whether interactions of any given species form a proper subset, nested within those of the more generalist ones; higher *wNODF* values indicating greater nestedness. Weighted modularity (*Qw′*) was computed using the DIRTLPAwb+ algorithm (Beckett, 2016) and indicates whether the network is structured into modules with denser within‐ than between‐module interactions (Olesen et al., 2007). *C, IE*, *H2*′ and *Qw*′ range from 0 to 1, with values closer to 1 indicating high network connectance, interaction evenness, specialisation and modularity, respectively. For *wNODF* and *Qw*′ metrics, we applied a ∆‐transformation by subtracting the mean value of random networks generated by a null model from empirical values. ∆*wNODF* and ∆*Qw*′ were used to correct deviations unrelated to network structure, enabling the comparison among networks with different sizes and connectance (Fründ et al., 2016). We used the Patefield algorithm to generate the null matrices, which preserves species richness and the total number of interactions (Patefield, 1981). All network metrics were assessed using the ‘bipartite’ package in R (Dormann et al., 2008; version 2.20).

Finally, we computed dissimilarities (i.e. β‐diversity) among plant–bee interaction networks across sampling periods. To preserve compositional differences among plots and flowering seasons, β‐diversity was assessed only among periods within the same plot and season, yielding 15 pairwise comparisons per plot and season (*n* = 360 comparisons across all periods, plots and seasons). To calculate interaction β‐diversity, we used the quantitative Ruzicka distance coefficient, which incorporates interaction frequencies among shared links between compared networks (Magrach et al., 2017). Three components of β‐diversity were calculated: β^OS^, reflecting changes in interaction composition among shared species (i.e. rewiring); β^ST^, representing interaction dissimilarities due to species turnover between sampling periods (i.e. turnover; Poisot et al., 2012); and β^WN^, quantifying the overall interaction dissimilarity between compared networks. β^ST^ and β^OS^ are additive components of total interaction β‐diversity, such that β^WN^ = β^ST^ + β^OS^. Values range from 0 to 1, with 1 indicating higher temporal β‐diversity in plant–bee interactions.

We fitted mixed models to test whether network structure and interaction composition differ among sampling periods. Network structure was analysed using separate models for each metric (pairwise interaction richness, *C*, *IE*, *H2*′, *wNODF* and *Qw*′), with the 2‐h sampling period as a categorical predictor. Interaction composition was evaluated by modelling β‐diversity, with β^WN^ as the response and the comparison between sampling periods as a categorical predictor, and by quantifying the contributions of β^OS^ and β^ST^. In all models, plot identity and season were included as crossed random effects to account for non‐independence arising from repeated observations across all plot–season combinations, and pairwise contrasts were assessed with Tukey tests using ‘emmeans’ R package (Lenth, 2016; version 1.10.3). Model details are given in Table S2.

### 2.3 Merging interactions into partial‐ and full‐day networks

Besides comparing compositional and structural properties of plant–bee networks among 2‐h sampling periods, we assessed how well single or combined periods represent the full daily network. Given our extensive sampling (*n* = 1440 h; 60 weeks), we used the pooled network across all sampling periods and plots (i.e. the full‐day network) as the benchmark of representativeness. This benchmark encompasses the daylight period when bees forage on flowers and integrates interactions recorded over 30 weeks spanning most of the flowering season. Quantitative evaluation of sampling completeness indicated that this full‐day network captured approximately 78% of the estimated total number of pairwise interactions (Figure S2). This approach ensured that the majority of plant–bee pairwise interactions were effectively sampled, thereby providing a robust basis for evaluating the representativeness of single (or combination of) sampling periods (i.e. partial‐day networks) in representing the structure and composition of plant–bee interaction networks.

We adopted a factorial approach to combine sampling periods, considering all possible combinations. At this stage, networks were constructed by pooling all plots to minimise spatial bias due to differences in plant composition among plots. To assess fine‐scale compositional and structural differences across sampling periods, we treated networks from each flowering season as independent sampling units, accounting for interannual variation in plant and bee composition. We generated 126 networks by merging all combinations of sampling periods within each flowering season (*n* = 62 partial‐day and one full‐day network per flowering season).

### 2.4 Assessing the structural and compositional representativeness of partial‐day networks

We subsequently estimated pairwise interaction richness, *C*, *IE*, *H2′*, *wNODF* and *Qw*′, to assess how representative each partial‐day network is relative to the full‐day network. Despite high sampling coverage, rare interactions may still have been missed due to the absence of particular species, potentially biasing the identification of the most representative partial‐day networks (see Dorado et al., 2011; Jordano, 2016). To account for this potential bias, we generated 1000 null matrices with the Patefield algorithm to compare the representativeness of each partial‐day network in delivering information of the properties of the network (e.g. pairwise interaction richness, *C*, *IE*, *H2′*, *wNODF* and *Qw*′; see Vizentin‐Bugoni et al., 2016 for a similar approach). We relaxed the network connectance in the generation of null matrices, which is fixed in other algorithms, such as ‘vaznull’ and ‘swap.web’, allowing for variability in the relative number of different pairwise interactions during our simulations. For pairwise interaction richness and *Qw*′, we worked only with 100 randomisations due to the extensive computation time required owing to the additional iterations those algorithms present. This procedure allowed us to compare combinations of sampling periods and select partial‐day networks whose topology most closely matched the full‐day network. To quantify similarity, we extracted z‐scores for each network metric using null matrices (*n* = 124,000 [or 12,400]: 1000 [or 100] for each of the 62 combinations per flowering season, excluding the full‐day network). Z‐scores measured deviations of each partial‐day network from the full‐day network mean. Partial‐day networks with z‐scores close to zero were therefore the most topologically similar to the full‐day network. We also computed the mean β^WN^ of plant–bee interactions between each partial‐day network and the full network within each flowering season. Because the full‐day network includes the largest number of unique interactions, this allowed us to identify which single or combined sampling periods best represent daily interaction assembly. Partial‐day networks with the lowest mean β‐diversity were therefore those capturing most existing plant–bee interactions.

### 2.5 Identifying network metrics less sensitive to varying sampling effort

Finally, to determine how many 2‐h sampling periods are needed to capture network properties across the full daylight span, we grouped partial‐day and full‐day networks (from one to six joint sampling periods) into six treatments representing sampling duration. We also treated network type (empirical vs. null) as a factor to assess how many periods are required to obtain metric values unlikely to arise by chance (Vizentin‐Bugoni et al., 2016). We then visually inspected how network metrics (pairwise interaction richness, *C*, *IE*, *H2*′, *wNODF*, *Qw*′) changed in empirical and null matrices as sampling periods were added. This analytical procedure allowed us to evaluate: (i) whether the estimated network metrics effectively preserve information about plant–bee interaction networks following the addition of sampling periods; and (ii) what network metric needs less sampling resolution to deliver scores that deviate from null expectations.

### 2.6 Intra‐ and inter‐seasonal consistency of daily interaction patterns

Because flowering and pollinator phenology vary markedly across the season in the Cerrado (Ballarin, Amorim, et al. 2026; Ballarin, Fidelis, et al., 2026), we evaluated whether patterns across daily sampling periods remained consistent under different seasonal contexts. We assessed temporal consistency in interaction frequency and pairwise interaction richness across sampling weeks, phenological blocks (early‐, mid‐ and late‐season), and flowering seasons, and repeated all core analyses—including network metrics, interaction β‐diversity and the representativeness of partial‐day networks relative to full‐day networks—across phenological blocks and flowering seasons. This allowed us to test whether seasonal turnover in species composition and interaction frequencies influenced comparisons among daily sampling periods. Full methodological details are provided in the Supplementary Methods.

## 3 RESULTS

We recorded 24,546 legitimate plant–bee interactions, of which more than half were detected during the mid‐morning period (i.e. 08:00–10:00 and 10:00–12:00), and this pattern was consistent across plots (Table 1). Sampling periods from 08:00–10:00 to 12:00–14:00 presented the highest richness of interacting plant and bee species (Table 1). Plant–bee interaction richness and the number of unique pairwise interactions restricted to a single sampling period reached their highest values around midday (i.e. from 08:00–10:00 to 12:00–14:00), with approximately 370 different plant–bee interactions, about 15–18% of which were unique to each single period (Table 1). However, interactions occurring at the beginning and end of the day (i.e. 06:00–08:00 or 16:00–18:00) still represented 20.35% of all unique pairwise interactions (Table 1).

**TABLE 1:** Summary metrics of interactions comprised in plant–bee networks encompassing each specific 2‐h time interval.

Sampling periods	Plant species richness	Bee species richness	Frequency of plant–bee interactions	% of interactions	Pairwise interaction richness	Unique pairwise interactions	% of unique pairwise interactions
6–8 h	74 (14.25 ± 2.86)	35 (12.15 ± 3.18)	3011 (125.46 ± 72.53)	12.27	236	24 (10.17%)	8.28
8–10 h	90 (23.21 ± 4.93)	47 (16.46 ± 3.75)	6130 (255.42 ± 155.36)	24.97	377	65 (17.24%)	22.41
10–12 h	92 (23.71 ± 4.89)	45 (16.61 ± 3.62)	7762 (323.42 ± 292.61)	31.62	369	57 (15.45%)	19.66
12–14 h	88 (22.37 ± 5.59)	45 (15.21 ± 3.50)	3971 (165.46 ± 89.25)	16.18	381	69 (18.11%)	23.79
14–16 h	78 (18.75 ± 4.77)	42 (14.04 ± 2.37)	2268 (94.50 ± 31.59)	9.24	329	40 (12.16%)	13.79
16–18 h	71 (14.01 ± 3.67)	41 (10.71 ± 2.68)	1404 (58.50 ± 27.61)	5.72	251	35 (13.94%)	12.07

*Note*: Mean ± SD in parentheses depicts values obtained for each plot. % values in parentheses represent the proportional score of unique pairwise interactions considering all inventories of plant–bee pairwise interactions within each sampling period.

### 3.1 Composition and structure of empirical networks across sampling periods within the day

All network metrics differed between sampling periods, although each metric showed distinct temporal patterns across the day, indicating that daily periods of data sampling influence the structure of plant–bee networks. Pairwise interaction richness was higher near midday (i.e. from 8:00 to 14:00; *χ* ^2^_5,139_ = 434.24; *R* ^2^‐_adjusted_ = 0.80; *p* < 0.01; Figure 1a). Network connectance (*C*) was higher in the period nearing dusk (i.e. 16:00–18:00; *χ* ^2^_5,139_ = 36.82; *R* ^2^‐_adjusted_ = 0.26; *p* < 0.01; Figure 1b). Interaction evenness (*IE*) and weighted nestedness (*∆wNODF*) increased consistently throughout the day, being significantly higher during sampling periods after midday (*IE*—*χ* ^2^_5,139_ = 76.62; *R* ^2^‐_adjusted_ = 0.85; *p* < 0.01; Figure 1c; ∆*wNODF*—*χ* ^2^_5,139_ = 103.82; *R* ^2^‐_adjusted_ = 0.60; *p* < 0.01; Figure 1e). Network‐wide specialisation degree (*H2′*) was higher in the early morning (i.e. 06:00–08:00; *χ* ^2^_5,139_ = 74.73; *R* ^2^‐_adjusted_ = 0.86; *p* < 0.01; Figure 1d). In contrast, the modularity (*Qw′*) of plant–bee networks decreased consistently throughout the day (*χ* ^2^_5,139_ = 50.49; *R* ^2^‐_adjusted_ = 0.60; *p* < 0.01; Figure 1f).

**FIGURE 1:**
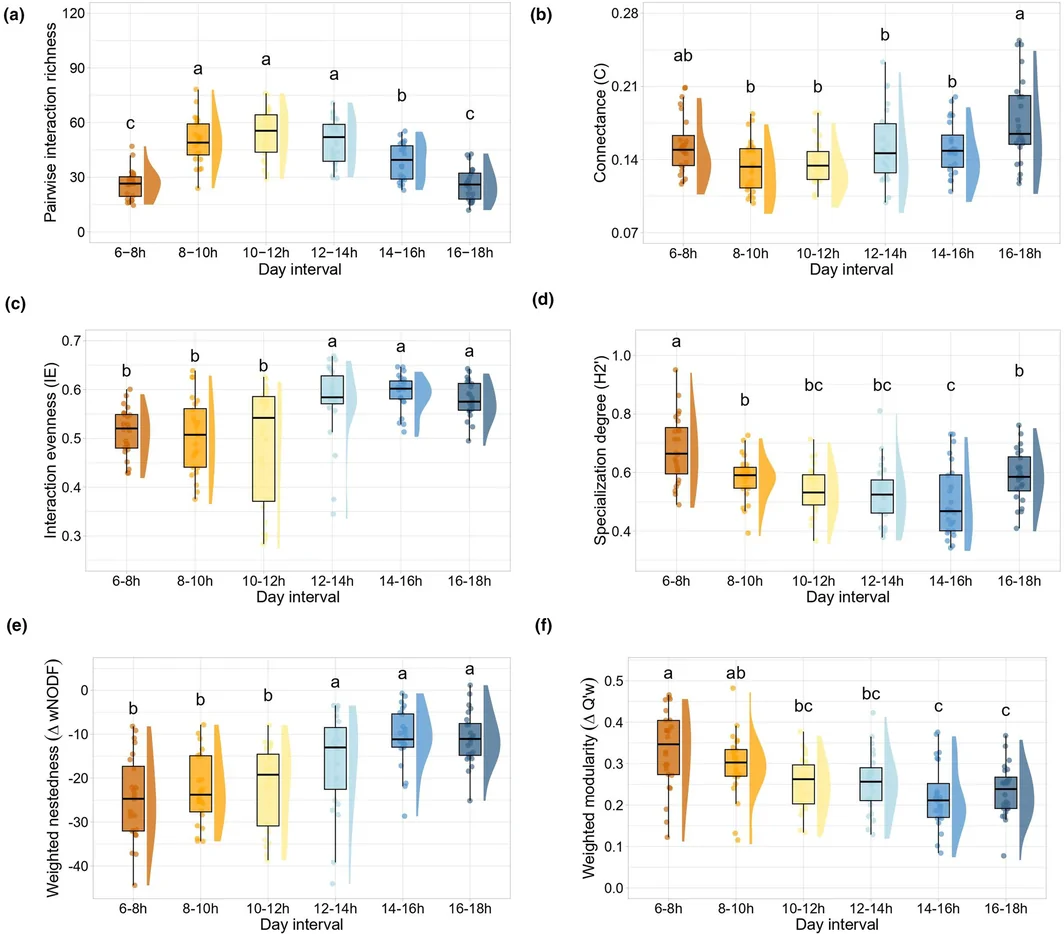
Network structure across daily sampling periods. Points depict the score of weighted network metrics extracted for each 2‐h sampling period in each plot and flowering season (*n* = 24 scores for each sampling period). Sampling periods occupying a central position within the day demonstrate consistently higher levels of pairwise interaction richness (a), but lower levels of connectance (b). From early morning to late‐afternoon (i.e. the orange‐blue continuum) networks exhibit progressively higher levels of interaction evenness (c) and nestedness (e), but lower levels of network specialisation (d) and compartmentalisation (f). Compact letter displays highlight significant differences in network metric scores following Tukey *post hoc* analyses.

Plant–bee interaction β‐diversity (i.e. β^WN^) was very different among sampling periods (*χ* ^2^_14,346_ = 488.62; *R* ^2^‐_adjusted_ = 0.98; *p* < 0.01). Notably, plant–bee interaction dissimilarity increased consistently with increasing temporal distance between sampling periods (Figure 2a). Furthermore, the lowest interaction β‐diversity occurred during sampling periods around midday (Figure 2a). Plant–bee interaction β‐diversity was driven more by interaction rewiring between shared species (i.e. β^OS^) than by the species turnover (i.e. β^ST^; Figure 2b).

**FIGURE 2:**
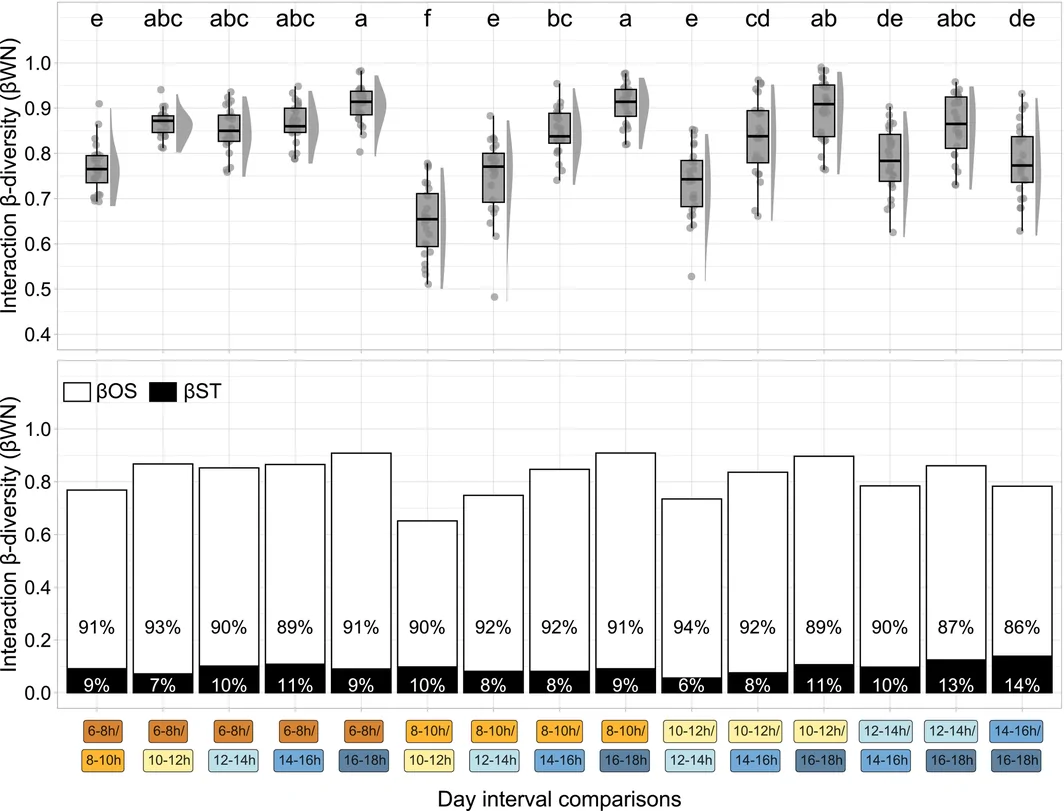
Interaction β‐diversity across daily sampling periods. Points represent pairwise comparisons among 2‐h sampling periods within the same plot and flowering season (*n* = 24 comparisons for each pair of sampling periods). Each boxplot represents one of all the possible comparisons between pairs of distinct sampling periods. Differences in overall interaction β‐diversity (i.e. β^WN^; a) increase with an extended time interval between compared sampling periods. Interaction rewiring (i.e. βOS; in white; b) is often more important than species turnover (β^ST^; in black; b) to explain the overall interaction β‐diversity. β^ST^ generally remains stable across comparisons of sampling periods but shows the lowest values when comparing periods occurring in the central hours of the day (e.g. 10‐12 h and 12‐14 h).

### 3.2 Assessing the structural and compositional representativeness of partial‐day networks

Increasing the number of sampling periods enhanced the potential for sampling a broader range of occurring plant–bee interactions. However, the ability of partial‐day networks to resemble the full‐day network in terms of structure varied across different combinations of sampling periods (Figure 3). While the pairwise interaction richness, *C*, *H2*′, *wNODF* and *Qw*′ became more similar to the full‐day network following progressive additions of 2‐h sampling periods (Figure 3a,b,d–f), *IE* did not follow the same trend (Figure 3c). Moreover, especially for interaction richness, *H2*′, *wNODF*, *Qw*′, the added value of sampling interaction around midday (i.e. from 08:00–10:00 to 12:00–14:00) was greater than focusing on sampling the extremes of the day (i.e. 06:00–8:00 and 16:00–18:00). This sampling interval alone accounts for 82.0% of all recorded interactions and 79.7% of all unique pairwise interactions (Table 1). In general, combinations of four 2‐h sampling periods, particularly from 08:00–10:00 to 14:00–16:00, were sufficient to capture network topology comparable to that obtained from continuous daylight monitoring. Despite that, covering the earliest and latest sampling period (i.e. 06:00–08:00 and 16:00–18:00) enhanced pairwise interaction richness, contributing to the completeness of recorded interactions.

**FIGURE 3:**
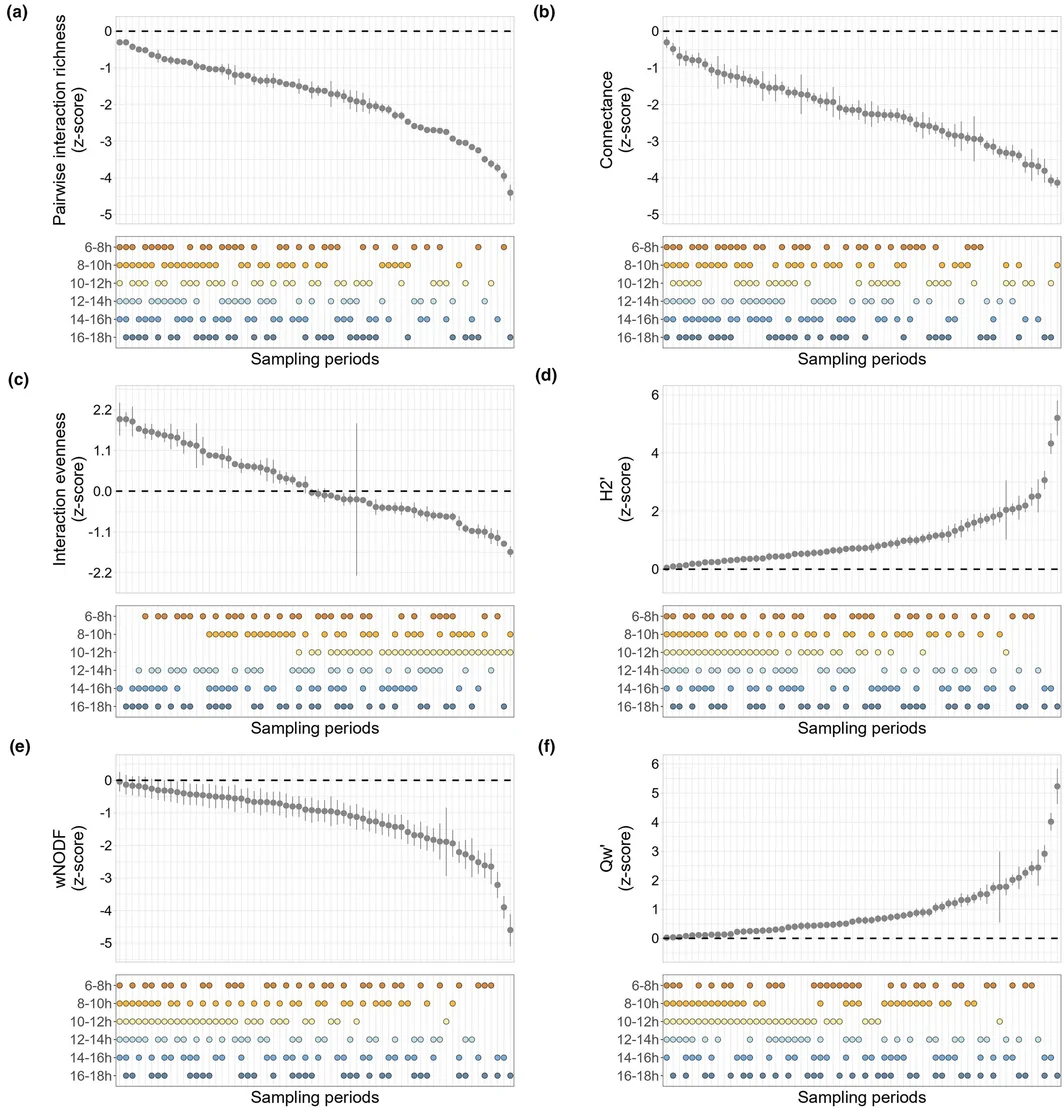
Optimal sampling periods resembling the full‐day network across 2‐h sampling combinations. Point ranges depict z‐score values obtained from null matrices for particular partial‐day networks (mean ± SD). Each panel contains 62 point ranges representing each partial‐day network. Partial‐day networks closer to the horizontal dashed line represent those that mostly align with the values of a specific network metric observed in the full‐day network. Circles below the graph are coloured following the orange‐blue continuum (6‐8 h: dark orange; 16–18 h: dark blue) and ordered according to the number of sampling periods that were merged to build the network.

Interaction composition dissimilarities (β^WN^) between the full‐ and the partial‐day networks generally decreased as additional 2‐h sampling periods were included (Figure 4). The best combinations for one to five periods showed a monotonic decrease in β^WN^, from a single central period (08:00–10:00; β^WN^ ≈ 0.75), to two (08:00–12:00; ≈0.45), three (08:00–14:00; ≈0.27), four (06:00–14:00; ≈0.15), and five periods (06:00–16:00; ≈0.05). Accordingly, combinations covering the central hours of the day (i.e. 08:00–14:00) consistently yielded partial‐day networks more similar to the full‐day network, outperforming other combinations with the same or even greater sampling effort that did not fully cover this central window (Figure 4). Additionally, sampling during the earliest period of the day proved to be more informative than sampling near dusk (Figure 4).

**FIGURE 4:**
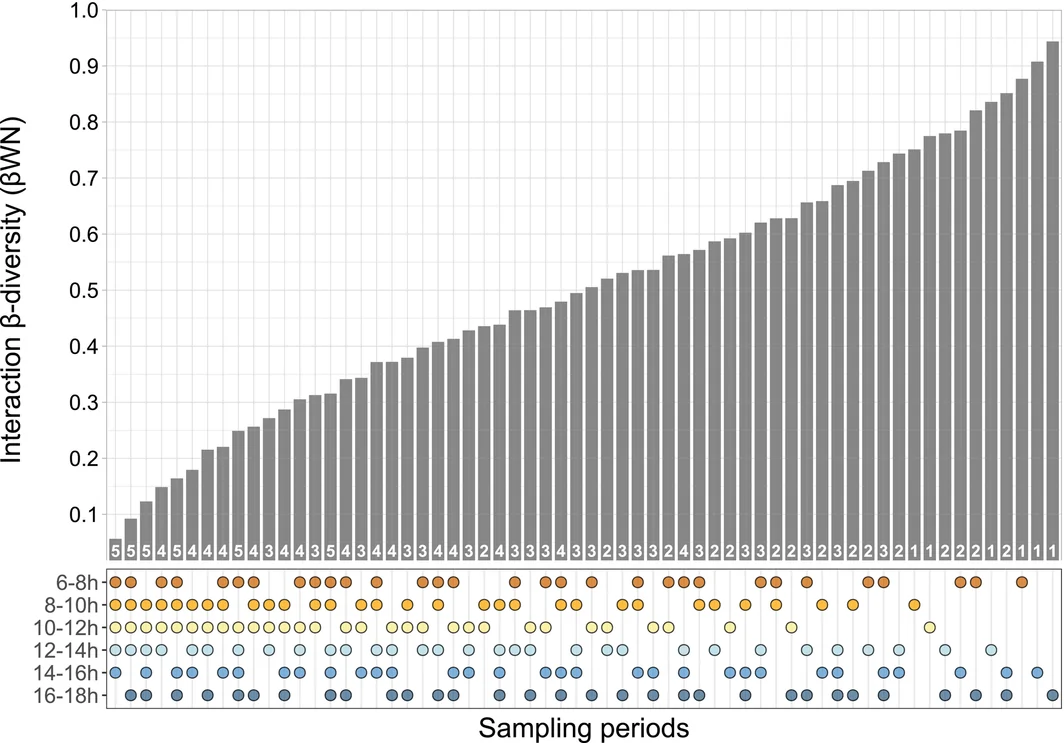
Plant–bee interaction β‐diversity among each partial‐ and the full‐day network. The bar plots represent the mean value of the overall β‐diversity of plant–bee interactions (i.e. β^WN^) among each partial‐day network and the full‐day network. Each bar corresponds to a partial‐day network, indicating which sampling periods were combined to create the interaction matrix. Circles below the graph are coloured following the orange‐blue continuum (6–8 h: dark orange; 16–18 h: dark blue) and ordered according to the number of sampling periods that were merged to build the network. The numbers within each bar denote the number of sampling periods that were merged.

Together, these results indicate that central daytime periods recover interaction composition close to the full‐day network, driving similarity in weighted metrics and overall network structure, whereas early‐morning and late‐afternoon intervals capture additional, presumably rare interactions.

### 3.3 Identifying network metrics less sensitive to varying sampling effort

The full‐day network was non‐nested, presented low connectance and exhibited moderate levels of specialisation and modularity (Figure 5). Although partial‐day networks increasingly resembled the full‐day network as more 2‐h periods were added, their topological properties still varied substantially with sampling effort (Figure 5). Following the addition of 2‐h sampling periods, pairwise interaction richness, connectance (*C*) and nestedness (*wNODF*) increased in both simulated and empirical networks (Figure 5a,b,e). Conversely, network specialisation (*H2′*) and modularity (*Qw′*) decreased in simulated networks but stabilised at intermediate values in empirical ones as sampling increased (Figure 5d,f). Interaction evenness (*IE*), in turn, became less variable and converged to intermediate values in both null and empirical networks (Figure 5c).

**FIGURE 5:**
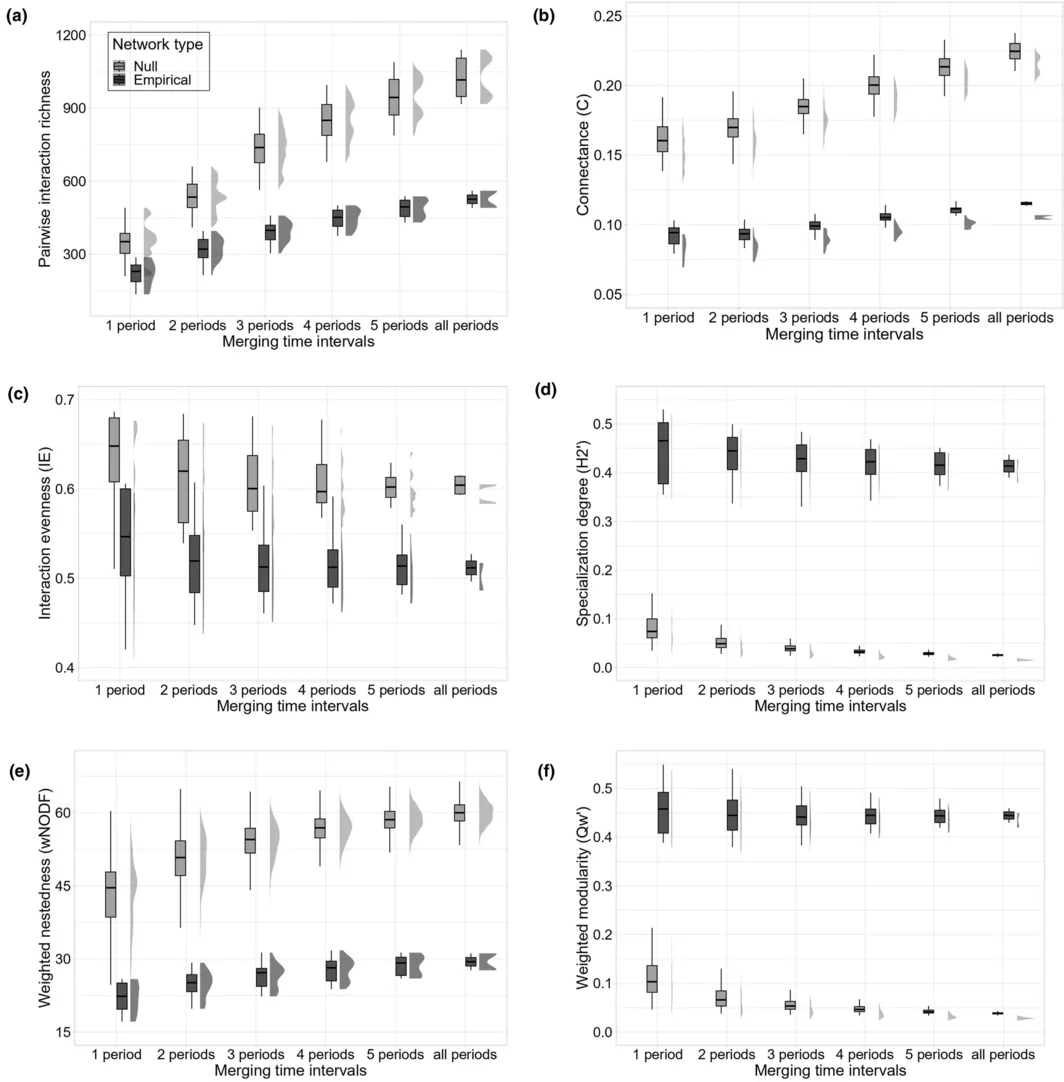
Projection of the network metrics extracted for null matrices for each partial‐ and full‐day network. In each graph, from the left to the right, boxplots illustrate the empirical (dark grey) or the expected (light grey) network metric scores for networks polling data on plant–bee interactions over one to six 2‐h sampling periods (i.e. the full‐day network). There is generally less variation in network metrics as the number of 2‐h sampling periods increases. While pairwise interaction richness (a), connectance (b) and network nestedness (e) tend to increase following the inclusion of additional daily sampling periods, the degree of network‐wide specialisation (d) and network modularity (f) tends to decrease. Intermediate scores of interaction evenness (c) are achieved with the addition of more 2‐h sampling periods. Non‐overlapping dark and light grey boxplots in each sampling period indicate that results from the empirical network are likely biologically meaningful (i.e. not obtained by chance).

The number of merged sampling periods influenced all studied network metrics (Figure 5). Pairwise interaction richness tended to be higher in null networks (Figure 5a). Furthermore, differences between null and empirical networks were evident across all sampling periods. Notably, *C*, *H2*′, *wNODF* and *Qw*′ differed significantly from randomised networks even under minimal sampling (i.e. single 2‐h periods; Figure 5b,d–f). For *IE*, at least four 2‐h sampling periods were required to achieve significant scores in empirical networks (Figure 5c).

### 3.4 Intra‐ and inter‐seasonal consistency of daily interaction patterns

Interaction frequency and pairwise interaction richness varied across weeks, phenological blocks and flowering seasons, but their relative distribution among 2‐h sampling periods remained consistent (Figures S3 and S4). Likewise, differences in network structure among periods were stable within and between flowering seasons (Figures S5 and S10), with only minor deviations during the early‐season block. Interaction dissimilarity also consistently increased with temporal distance between periods across phenological blocks (Figure S11; Tables S3 and S4). Similarly, assessments of partial‐day network representativeness were consistent across seasons and phenological blocks (Figure S12), with midday periods showing the lowest dissimilarity relative to full‐day networks. Thus, although seasonal phenological turnover affected overall interaction magnitude, it did not explain differences among 2‐h daily sampling periods.

## 4 DISCUSSION

Empirically sampled ecological interaction inventories, as any result of a sampling protocol, often exhibit a subset of the actual pairwise species‐species interactions occurring in nature (Chacoff et al., 2012; Jordano, 2016). One of the reasons for such limitation arises from selecting a specific temporal window that can capture the fraction of interactions only occurring within that timeframe. However, it is common to extrapolate observed patterns of ecological interactions from a specific temporal window to infer underlying processes encompassing interactions in unsampled windows. For instance, many recent studies worldwide have focused on sampling plant–bee interactions during specific daylight hours to optimise effort and cost while maximising interaction records (see Hachuy‐Filho et al., 2020; Burkle et al., 2022 as examples). However, they might implicitly assume that the findings from this limited timeframe reflect patterns across the entire daylight period. In this study, we demonstrate that sampling plant–bee interactions at different times throughout the day leads to considerable variability in plant–bee network composition and structure. Through this method‐directed analysis, we demonstrate that strategically balancing sampling efforts across different periods of the day ensures datasets of plant–bee interactions capable of reliably reflecting the structure and properties of full‐day sampled networks. The full‐day network was used as a benchmark because it represents the most complete approximation of plant–bee interactions occurring within the daylight activity window, which is the relevant temporal scale over which these interactions take place.

Our results show that restricting sampling to specific portions of the day captures dominant interaction patterns but misses many unique and rare interactions, altering both network composition and inferred structure. This has important implications for ecological inference, as studies comparing network structure across ecological gradients (e.g. latitude, elevation or habitat type) may attribute differences in metrics, such as specialisation or nestedness, to environmental drivers when they instead reflect differences in daily sampling windows. By comparing partial‐day networks to a full‐day benchmark, our approach provides a more robust assessment of how restricted temporal sampling may bias inferences about ecological network structure and composition. Thus, similarly to studies showing that time‐sensitive sampling must be accounted for at broader temporal scales (e.g. CaraDonna et al., 2021; Olesen et al., 2010; Schwarz et al., 2020), our results demonstrate that sampling only part of the day may not reliably represent daily interactions, with consequences for the representation of network structure.

### 4.1 Composition and structure of empirical networks across sampling periods within the day

Network structure varied consistently among sampling periods within the day. Specialisation and modularity were higher in early‐morning periods, whereas nestedness and interaction evenness increased after midday. Interaction richness and frequency peaked during midday periods, with lower values observed at the beginning and end of the day. This pattern suggests a shift from more modular and specialised interaction sets in the early morning to more generalised and hierarchically organised patterns later in the day. The higher connectance near dusk is consistent with fewer plant species remaining in bloom, forcing pollinators to concentrate on a reduced set of resources and generating a denser web of interactions. Accordingly, the species‐turnover component explained a larger yet still minor fraction of overall β‐diversity near dusk, likely due to flower closure in some plant species. These findings imply that resource utilisation patterns undergo significant changes from dawn to dusk, primarily due to the rewiring of interactions, albeit influenced to some degree by species turnover within the day (Ratoni et al., 2025; but see Nagano, 2023). The dynamical rewiring of interactions throughout the day and the strong contribution of interaction richness and frequency around midday periods are likely to play a key role in shaping the daily structural variation of plant–bee ecological networks.

Plant and bee life‐history traits shape interaction patterns across the day. On the plant side, flowering phenology and resource availability strongly influence pollinator use (Baldock et al., 2011; Herrera, 1990). Some species open or close flowers at specific times, creating forbidden links for pollinators active outside those periods (Fründ et al., 2011), boosting interaction turnover. Moreover, flowers often show daily peaks in nectar production and pollen presentation, prompting shifts in foraging strategies and rewiring interactions among visitors (Ballarin et al., 2022; Štenc et al., 2023). On the bee side, foraging activity varies among species according to morphological and physiological traits that mediate responses to wind, temperature and light (Herrera, 1990, 2024; Polatto et al., 2014; Willmer & Stone, 2004), driving interaction turnover. Moreover, changes in floral availability at fine temporal scales lead bees to adjust their foraging, increasing interaction rewiring (Herrera, 1990; Polatto et al., 2014; Nagano, 2023; Arrowsmith et al., 2025; Arroyo‐Correa, 2025). Such temporal dynamics in floral availability help explain why specialisation and modularity are higher in the early morning, when interactions are dominated by large‐bodied and more specialised bees (e.g. *Centris* and *Epicharis*; see Table S1) that tolerate milder climatic conditions and often concentrate their foraging on a single plant species offering resources of restricted use (e.g. oil). As the day progresses towards midday, the increasing number of simultaneously open flowers expands resource availability, reducing specialisation and gradually increasing nestedness as interactions become structured by a more hierarchical and competitive use of resources. Later in the day, flower closure and resource depletion further reduce resource availability, leading to higher connectance and interaction evenness, and reinforcing nestedness. Thus, the timing of flower opening, the dynamics of floral reward availability and diel patterns of bee activity likely modulate visitation to co‐flowering plants and shape network structure throughout the day.

All this suggests that early‐morning periods disproportionately capture interactions involving more specialised species and well‐defined modules, whereas midday and late‐day networks increasingly reflect the activity of generalist species and the progressive concentration of interactions on fewer floral resources. Consequently, the time‐of‐day emphasised in empirical studies will shape which facets of network organisation are most clearly expressed, from specialised and modular structures to the broader structure of resource competition.

These temporal shifts in interaction structure have important implications for conservation and ecological inference. Temporally restricted sampling may overrepresent interactions dominating specific periods while underrepresenting those contributing to network structure at other times. Consequently, estimates of connectivity, nestedness or interaction distribution may reflect the sampling window rather than the integrated diurnal structure of the network. This bias can also affect conservation prioritisation by emphasising taxa or links disproportionately represented during peak sampling periods (Ballarin et al., 2024; Ulrich & Peters, 2023) while overlooking interactions important in other daily windows. In systems where interaction structure varies across the day, this may lead to incomplete representations of network organisation and suboptimal conservation decisions.

### 4.2 Assessing the structural and compositional representativeness of partial‐day networks

Beyond documenting daily changes in empirical plant–bee interaction networks, our chronologically structured sampling protocol allowed us to evaluate how different combinations of 2‐h periods perform in reconstructing network structure under realistic sampling schedules. Although sampling throughout the entire day provides the most complete description of the system (Baldock et al., 2011), logistic and funding limitations often make this unfeasible in practice (Hegland et al., 2010).

Our results show that strategically combining daily sampling windows can efficiently approximate full‐day network structure. Networks assembled from four 2‐h periods consistently achieved low interaction turnover (β^WN^ <0.2) relative to the full‐day network, while recovering up to ~80% of all pairwise interactions and ~82% of interaction frequencies. Combinations dominated by central daytime hours maximised interaction richness and, critically, reproduced interaction frequencies close to those of the full‐day network, which explains their high resemblance in weighted network metrics. This reflects the peak of bee activity, when most bee‐pollinated plants are simultaneously open and rewarding (Nagano, 2023). In contrast, early‐morning and late‐afternoon periods, although contributing fewer interactions, add a disproportionate number of unique links, presumably rarer and temporally restricted, thereby enriching the pool of recorded pairwise interactions.

Still, sampling design and metric choice should be aligned with study goals. If the objective is to characterise network structure, interaction asymmetries or identify core species involved in frequent interactions, central daytime windows are most efficient; if the goal is to maximise interaction diversity, detect specialised or rare links and better resolve modular structure, distributing sampling across the day, especially including early‐morning periods, is likely ideal, as it introduces interactions absent from other periods but frequent enough to influence network structure.

### 4.3 Identifying network metrics less sensitive to varying sampling effort

Frequency‐based network metrics consistently captured network structure and showed predictable trends with the addition of sampling periods (Vizentin‐Bugoni et al., 2016). While *IE* and interaction richness exhibited higher variability across combinations, *H2*′, *wNODF* and *Qw*′ converged rapidly towards full‐day values when sampling was concentrated in the central daylight hours. Overall, increased sampling effort increased nestedness and connectance, whereas intermediate levels of specialisation and modularity emerged as periods were added. Importantly, empirical values of *C*, *H2*′, *wNODF* and *Qw*′ consistently departed from null expectations, indicating that these metrics capture biologically structured patterns even under temporally restricted sampling.

Our results show that network metrics differ in their sensitivity to restricted temporal sampling. Under limited sampling windows, weighted metrics provide more reliable and stable estimates of network structure, especially when sampling is concentrated in periods that accumulate higher interaction richness and frequencies. This is because metrics, such as *H2*′, *wNODF*, *Qw*′ and *C* readily depart from null expectations and show comparatively smaller variation as additional sampling periods are added. In contrast, pairwise interaction richness and interaction evenness are more sensitive to sampling effort and only stabilise and consistently depart from null models after a larger fraction of the day has been sampled. Therefore, when temporal coverage is necessarily restricted, weighted metrics are more appropriate for structural inference, whereas characterising the full pool and diversity of interactions requires distributing sampling effort across the day.

### 4.4 Intra‐ and inter‐seasonal consistency of daily interaction patterns

Although temporal variation in plant–pollinator network structure across the flowering season is well documented (e.g. Alarcón et al., 2008; Ballarin, Amorim, et al. 2026; Ballarin, Fidelis, et al., 2026; CaraDonna et al., 2017; Resasco et al., 2021), our results show that intra‐daily variation is also a consistent component of network organisation. Some reduction in this consistency occurs early in the season, when lower species richness and cooler temperatures increase variability in interactions and amplify short‐term ecological constraints. Despite this, the overall diel signature remains detectable across the season, reinforcing that daily processes are embedded within seasonal dynamics. Consequently, intra‐daily variation should be explicitly considered in network studies, as broader‐scale patterns attributed to seasonal or interannual processes may partly reflect differences in sampling time‐of‐day. This has direct implications for cross‐study comparisons, particularly when networks are sampled across different flowering periods without standardised daily sampling windows, potentially confounding temporal scales in inferred network structure.

### 4.5 A general framework for daily sampling of time‐sensitive interactions

While increasing sampling effort is time‐ and labour‐intensive, monitoring ecological interactions is increasingly critical in systems undergoing rapid environmental change (Jordano, 2016). Here, rather than proposing a universal protocol, we outline a general framework to evaluate how the choice of daily sampling windows affects the representativeness of interaction networks (Figure 6). The main idea is that missing interactions are not necessarily recovered by increasing effort within a restricted time window, but often by expanding or repositioning the sampling window itself.

**FIGURE 6:**
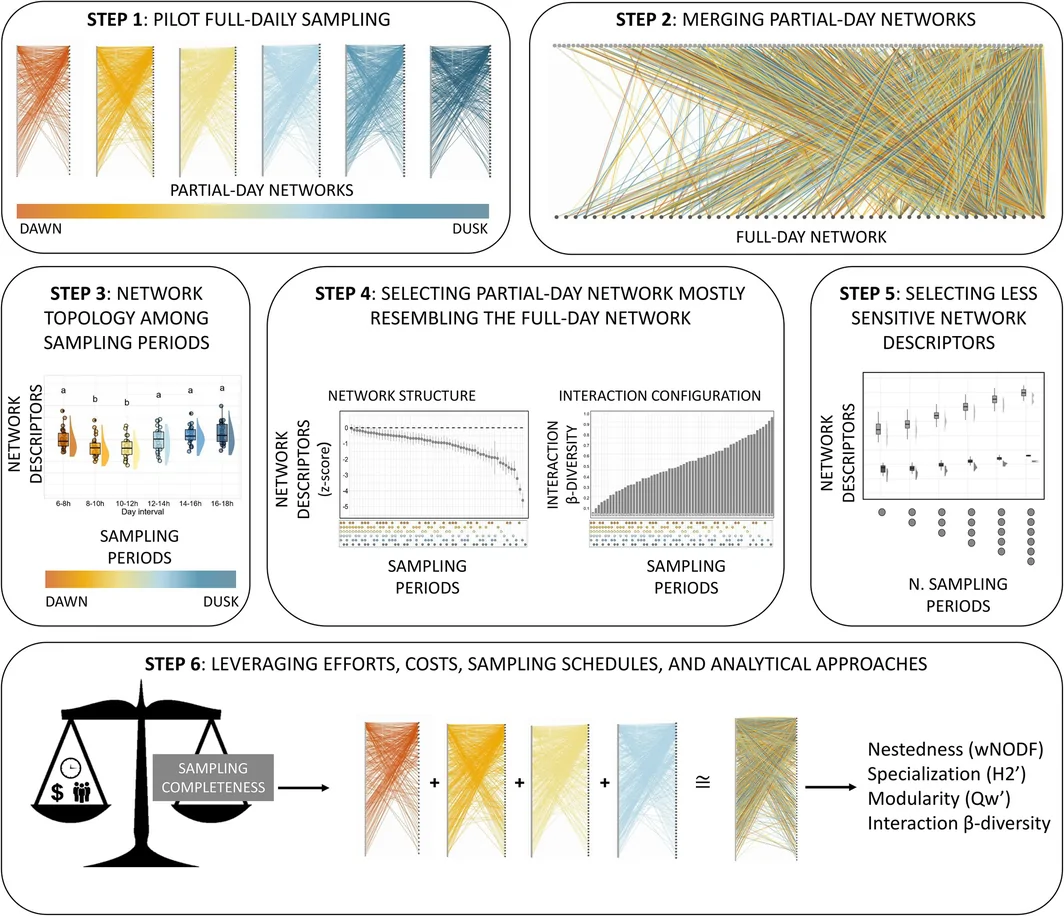
Framework for the daily sampling of plant–bee interaction networks. A step‐by‐step routine is suggested to inspect whether the structure and configuration of plant–bee interaction networks vary throughout the day and to determine the best combination of sampling periods to build partial‐day networks closely resembling the full‐day network.

The approach consists of conducting pilot samplings across the main daily windows of activity, building time‐specific networks, and then combining them to generate partial‐ and full‐day networks (Hegland et al., 2010). Partial‐day networks can then be evaluated according to how well they approximate the full‐day network in terms of structure and interaction configuration, and network metrics can be screened for their sensitivity to incomplete temporal coverage (Vizentin‐Bugoni et al., 2016). This allows researchers to identify (i) the minimum effort needed to obtain a representative network, (ii) how this effort should be distributed across the day, and (iii) which descriptors are more robust to temporal subsampling. For example, when full‐day sampling is unfeasible due to long daylight periods or logistical constraints, the day can be divided into discrete time slices to identify combinations that best approximate the full‐day network.

Importantly, the most informative sampling window will be context‐dependent. Systems may differ markedly in their diel dynamics, and, in some cases, sampling during the periods of highest interaction activity will disproportionately increase network representativeness, likely because these periods concentrate not only interaction frequency but also interacting species richness. In other ecological contexts (e.g. temperate or species‐poor systems), plant–pollinator diel dynamics may follow different patterns. Nevertheless, even in such cases, the daily interaction window can still be partitioned into meaningful intervals tailored to identify the most informative periods. Although our discussion is framed around plant–bee interactions, the same logic applies whenever interactions are structured in temporal or spatial slices (e.g. plots, months, seasons or years) and is particularly relevant for conservation‐oriented studies and comparative syntheses that implicitly assume that a locally sampled network is representative of the underlying interaction system.

## 5 CONCLUSION

Daily changes in pairwise plant–bee interactions have a significant signal in plant–bee network structure, which implies that different sampling schedules may yield different, somehow biased, network properties. Still, we demonstrate that employing a comprehensive sampling design that encompasses the period of higher plant availability and bee activity might yield results resembling a network covering the entire daylight span. Although sampling plant–bee interactions spanning all the day might be demanding, future studies should (i) draw sampling schedules aiming to track daily variations in plant–bee pairwise interactions or (ii) explicitly recognise that results coming from limited sampling periods should ideally not be extrapolated to broader temporal daily windows, as networks built in different daily timeframes may manifest significant topological disparities.

## AUTHOR CONTRIBUTIONS

Caio S. Ballarin and Jorge Isla conceived the ideas. Caio S. Ballarin designed the methodology. Caio S. Ballarin and Leandro Hachuy‐Filho collected the data. Caio S. Ballarin, Jorge Isla, Elena Quintero, Blanca Arroyo‐Correa and Pedro Jordano analysed the data and validated the results. Caio S. Ballarin led the writing of the manuscript. All authors contributed critically to the drafts and gave final approval for publication.

## CONFLICT OF INTEREST STATEMENT

There is no conflict of interest to disclose.

Our study brings together authors from two different countries (and continents), including scientists based in the country where the study was carried out. Whenever relevant, literature published by scientists from the region was cited; efforts were made to consider relevant work published in the local language.

## Supporting information


**Figure S1.** Sampling completeness profile of the daily sampling periods.
**Figure S2.** Sampling completeness of the full‐day plant–bee interaction network.
**Figure S3.** Weekly variation in interaction intensity among daily sampling periods.
**Figure S4.** Intra‐seasonal variation in interaction intensity among daily sampling periods.
**Figure S5.** Pairwise interaction richness across phenological blocks and flowering seasons.
**Figure S6.** Connectance across phenological blocks and flowering seasons.
**Figure S7.** Interaction evenness across phenological blocks and flowering seasons.
**Figure S8.** Network‐wide specialisation degree across phenological blocks and flowering seasons.
**Figure S9.** Weighted nestedness across phenological blocks and flowering seasons.
**Figure S10.** Weighted modularity across phenological blocks and flowering seasons.
**Figure S11.** Intra‐seasonal phenological variation interaction β‐diversity among daily sampling periods.
**Figure S12.** Plant–bee interaction β‐diversity among each partial‐ and the full‐day network and across phenological blocks and flowering seasons.
**Table S1.** List of bee taxa recorded in the study and their interaction patterns.
**Table S2.** Mixed‐model specifications used to test temporal variation in network structure and pairwise interaction turnover across sampling periods.
**Table S3.** Full model selection table (AICc) for testing whether intra‐seasonal blocks (early‐, mid‐ and late‐season) modify daily patterns of interaction β‐diversity (β^WN^).
**Table S4.** Hierarchical variance partitioning of interaction β‐diversity (β^WN^) across intraday and seasonal temporal scales.

## Data Availability

Data are available from the Figshare Repository: https://doi.org/10.6084/m9.figshare.32744535 (S. Ballarin, Isla, et al., 2026).
