# Supplementary material for: Optimising daily sampling of plant–bee interaction networks
Source: J Anim Ecol. 2026 Jul 15;95(10):1903–18. doi: 10.1111/1365-2656.70309 (PMC13643181; doi:10.1111/1365-2656.70309)
Supplement: Supplementary file 1 [file JANE-95-1903-s001.docx]

**Supplementary material**

**Optimising daily sampling of plant–bee interaction networks**

Caio S. Ballarin^1,2,3*^, Jorge Isla^3^, Blanca Arroyo-Correa^3^, Elena Quintero^3,4^, Leandro Hachuy-Filho^1,5^, Felipe W. Amorim^1,2,5a^, Pedro Jordano^3,4a^

^1^ Laboratório de Ecologia da Polinização e Interações – LEPI, Departamento de Biodiversidade e Bioestatística, Instituto de Biociências, Universidade Estadual Paulista “Júlio de Mesquita Filho” (UNESP), Rua Prof. Dr. Antonio Celso Wagner Zanin, CEP 18618-689, Botucatu, São Paulo State, Brazil

^2^ Programa de Pós-graduação em Biologia Vegetal, Instituto de Biociências, Universidade Estadual Paulista “Júlio de Mesquita Filho”

^3^ Integrative Ecology Group, Estación Biológica de Doñana, EBD-CSIC, Av. Américo Vespucio 26, E-41092 Sevilla, Spain

^4^ Departamento de Biología Vegetal y Ecología, Universidad de Sevilla, Av. Reina Mercedes, E-41012 Sevilla, Spain

^5^ Programa de Pós-graduação em Zoologia, Instituto de Biociências, Universidade Estadual Paulista “Júlio de Mesquita Filho”

^a^ co-senior authors

**Correspondence:** Caio S. Ballarin

E-mail address: [csballarin@gmail.com](mailto:csballarin@gmail.com)

**Content**

**Supplementary Methods.** Seasonal analyses.

**Figure S1.** Sampling completeness profile of the daily sampling periods.

**Figure S2.** Sampling completeness of the full-day plant–bee interaction network.

**Figure S3.** Weekly variation in interaction intensity among daily sampling periods.

**Figure S4.** Intraseasonal variation in interaction intensity among daily sampling periods.

**Figure S5.** Pairwise interaction richness across phenological blocks and flowering seasons.

**Figure S6.** Connectance across phenological blocks and flowering seasons.

**Figure S7.** Interaction evenness across phenological blocks and flowering seasons.

**Figure S8.** Network-wide specialization degree across phenological blocks and flowering seasons.

**Figure S9.** Weighted nestedness across phenological blocks and flowering seasons.

**Figure S10.** Weighted modularity across phenological blocks and flowering seasons.

**Figure S11.** Intra-seasonal phenological variation interaction β-diversity among daily sampling periods.

**Figure S12.** Plant–bee interaction β-diversity among each partial- and the full-day network and across phenological blocks and flowering seasons.

**Table S1.** List of bee taxa recorded in the study and their interaction patterns.

**Table S2.** Mixed-model specifications used to test temporal variation in network structure and pairwise interaction turnover across sampling periods.

**Table S3**. Full model selection table (AICc) for testing whether intraseasonal blocks (early-, mid-, and late-season) modify daily patterns of interaction β-diversity (β^WN^).

**Table S4.** Hierarchical variance partitioning of interaction β-diversity (β^WN^) across intraday and seasonal temporal scales.

**References.**

**Supplementary Methods — Seasonal analyses**

*Seasonal consistence of daily interaction patterns*

Interaction frequency and richness

Because flowering and pollinator phenology vary markedly across the season in the Cerrado (Ballarin et al. 2026), we conducted additional analyses to evaluate whether intra- and inter-seasonal variation could bias comparisons among daily sampling periods.

First, we visually explored the logarithm of (i) interaction frequency and (ii) pairwise interaction richness for each daily sampling period across all sampling weeks in both flowering seasons (Figure S3). This exploratory analysis aimed to assess whether the relative contribution of each daily period to total interaction activity and interaction richness changed across weeks or between seasons, which would indicate a potential seasonal permutation in the dominance of daily periods.

Second, because sampling was conducted weekly over 30 weeks in each flowering season, we divided the sampling period into three phenological blocks: early-, mid-, and late-season, which broadly reflect seasonal changes in solar radiation, temperature, and humidity. We then visually explored interaction frequency and pairwise interaction richness among daily periods within each of these three seasonal blocks to assess whether daily patterns were consistent across the season (Figure S4).

Network structure

Third, we quantified network structure across sampling periods by constructing interaction networks for each combination of flowering season, phenological block (early-, mid-, and late-season), plot, and 2-h sampling interval. From these networks, we calculated pairwise interaction richness, connectance (C), interaction evenness (IE), network-level specialisation (H2’), weighted nestedness (wNODF), and weighted modularity (Qw’). For nestedness and modularity, we used Δ-transformed values (ΔwNODF and ΔQw’) based on null model expectations.

Detailed descriptions of these metrics are provided in the main text (Materials and Methods).

We then visually inspected the distribution of these network metrics across sampling periods, flowering seasons, and phenological blocks (early-, mid-, and late-season) to assess how network structure varies within the day and if this pattern is consistent within and across flowering seasons (Figure S5 – S10).

Interaction β-diversity

Fourth, to formally test whether seasonal variation in species occurrence could influence interaction turnover and bias our β-diversity results, we repeated the interaction network analyses separately for each phenological block. For each block, we built interaction networks for each plot, flowering season, and daily sampling period by aggregating all interactions recorded within that block. Within each block, and following exactly the same approach described in the main text, we calculated overall interaction β-diversity (β^WN^) among daily sampling periods (Figure S11a).

We then fitted generalized linear mixed models with beta error distribution, using β^WN^ as the response variable and including the interaction between daily period comparison (e.g., 06:00-08:00h *versus* 08:00-10:00h), and phenological block (i.e., early-, mid-, late-season), as well as flowering season (i.e., first and second flowering season), as fixed effects, and plot identity as a random effect. Model selection was performed using the dredge function in the *MuMIn* package, and models were ranked using AICc (Table S3). If intra-seasonal phenological variation strongly influenced the daily patterns reported in the main text, the best-supported models would be expected to include interactions between daily period and phenological block.

In addition, to quantify the relative contribution of intraday *versus* seasonal processes to interaction β-diversity, we adopted a hierarchical variance decomposition approach based on a sequence of generalized linear mixed models with beta error distribution. We first fitted a null model including only plot identity as a random effect. We then added daily period comparison as a fixed effect to test how much variance is explained solely by intraday temporal structure. Next, we added phenological block (early-, mid-, late-season) to account for intra-seasonal variation in species composition. Finally, we fitted the full model including flowering season and all interactions among factors. For each model, we computed marginal R² (Nakagawa & Schielzeth, 2013) and quantified the incremental increase in explained variance (ΔR²) between successive models (Table S4). This approach allows disentangling the variance attributable to intraday temporal organization from that associated with seasonal phenological turnover.

Finally, for each phenological block (early, mid, late), we calculated Spearman correlations between temporal distance among daily periods and interaction β-diversity (β^WN^) (Figure S11b-d). Similar magnitude and direction of these correlations across blocks would indicate that the variation in interaction dissimilarity across daily sampling periods is consistent even when controlling for intra-seasonal phenological variation.

Representativeness of partial-day networks in resembling the full day network

Fifth, to assess how well different sampling periods capture daily interaction patterns, we compared partial-day networks (i.e., networks constructed from single or combined 2-h intervals) with a full-day reference network. For each flowering season and phenological block, the full-day network was built by pooling all sampling periods and used as a benchmark.

We generated all possible combinations of sampling periods and constructed the corresponding partial-day networks. Representativeness was evaluated by measuring interaction β-diversity (β^WN^) between each partial-day network and the corresponding full-day network, as described in the main text (Materials and Methods).

Lower β-diversity values indicate that a given sampling combination more closely approximates the full set of daily interactions. We visually inspected these patterns to identify which sampling periods or combinations best represent daily interaction structure across flowering seasons and phenological blocks (Figure S12).

All graphical and statistical results of these analyses are presented in Figures S3–S12 and Table S3 and S4.

**Figure S1. Sampling completeness profile of the daily sampling periods.** The completeness of plant–bee pairwise interaction inventories for each sampling period, pooling interaction data from all plots (a). Empirical plant–bee interaction richness reached a modal value of 72.33 ± 3.27% compared to what could be expected if all pairwise interactions were sampled. Comparisons of sampling completeness among 2-hour sampling periods within each plot (b) revealed slight differences, with late afternoon (i.e., 16-18h) showing the lowest score.

**Figure S2. Sampling completeness of the full-day plant–bee interaction network**. Rarefaction and extrapolation curve of interaction richness for the full-day network, built by combining all sampling periods and plots. The solid line represents the rarefaction curve based on the observed data, and the dashed line represents the extrapolation beyond the observed sampling effort. The shaded area indicates 95% confidence intervals. The black dot marks the observed sampling effort. Sampling completeness was quantified as the ratio between the number of observed interactions and the estimated total interaction richness, indicating that approximately 78% of the estimated plant–bee pairwise interactions were captured by the full-day network.

**Figure S3. Weekly variation in interaction intensity among daily sampling periods.** Panels (a) and (c) show the temporal dynamics of log_10_-transformed total interaction frequency and log_10_-transformed total interaction richness, respectively, across the weeks of sampling within the first and second flowering seasons. Panels (b) and (d) show the corresponding relative interaction frequency and relative interaction richness, expressed as the proportion of all interactions recorded in each week that occurred in each 2h -daily period (e.g., 6-8h, 8-10h). Although both interaction frequency and richness exhibit marked intraseasonal variation, reflecting changes in plant and pollinator occurrence associated with their phenologies, the relative ranking among daily periods remains largely consistent through time: periods with higher interaction frequency and richness tend to remain higher across weeks, whereas periods with lower values remain consistently lower. This indicates that, despite strong intraseasonal fluctuations in the overall availability of interactions, the structure of interaction allocation among daily periods is relatively stable. Together, these results show that intraseasonal phenological variation affects the magnitude of interactions but does not substantially modify the comparative differences among daily sampling periods.

**Figure S4. Intraseasonal variation in interaction intensity among daily sampling periods.** Panels (a) and (c) show log_10_-transformed total interaction frequency and log_10_-transformed total interaction richness, respectively, summarized for each phenological block (early, mid, and late-season) in the first and second flowering seasons. Panels (b) and (d) show the corresponding relative interaction frequency and relative interaction richness, expressed as the proportion of all interactions recorded within each phenological block that occurred in each daily period. In contrast to the week-by-week representation, this block-level aggregation further emphasizes that daily periods consistently differ in their interaction intensity, with periods showing higher frequency and richness remaining higher across phenological blocks, and periods with lower values remaining consistently lower. This indicates that, even when accounting for intraseasonal shifts in plant and pollinator occurrence associated with phenology, the allocation of interactions among daily periods is highly stable across the flowering season. Thus, phenological turnover modifies the overall magnitude of interactions but does not alter the relative differences among daily sampling periods.

**Figure S5. Pairwise interaction richness across phenological blocks and flowering seasons**. Boxplots with accompanying violin plots depict the distribution of pairwise interaction richness across 2‑h sampling intervals (6–8h, 8–10h, 10–12h, 12–14h, 14–16h, 16–18h), color‑coded along the orange–blue continuum to represent progression from early morning to late afternoon. Data are partitioned by flowering season (Season 1: 2021/2022; Season 2: 2022/2023) and subdivided into early, mid, and late phenological blocks (10 weeks each). While seasonal structuring introduces some variation, the dominant signal remains the within‑day dynamics: pairwise interaction richness is consistently higher near midday, revealing a temporal pattern that parallels the intra‑day trends described in Figure 1a.

**Figure S6. Connectance across phenological blocks and flowering seasons.** Boxplots with accompanying violin plots depict the distribution of connectance (C) across 2‑h sampling intervals (6–8h, 8–10h, 10–12h, 12–14h, 14–16h, 16–18h), color‑coded along the orange–blue continuum to represent progression from early morning to late afternoon. Data are partitioned by flowering season (Season 1: 2021/2022; Season 2: 2022/2023) and subdivided into early, mid, and late phenological blocks (10 weeks each). Although seasonal structuring introduces some deviations, particularly during the early‑season, the dominant signal remains the within‑day variation: C values tend to peak at both dawn and dusk, revealing a temporal pattern broadly aligned with that described in Figure 1b.

**Figure S7. Interaction evenness across phenological blocks and flowering seasons.** Boxplots with accompanying violin plots depict the distribution of interaction evenness (IE) across 2‑h sampling intervals (6–8h, 8–10h, 10–12h, 12–14h, 14–16h, 16–18h), color‑coded along the orange–blue continuum to represent progression from early morning to late afternoon. Data are partitioned by flowering season (Season 1: 2021/2022; Season 2: 2022/2023) and subdivided into early, mid, and late phenological blocks (10 weeks each). Although seasonal structuring introduces some deviations, particularly during the early‑season, the dominant signal remains the within‑day variation: IE increases progressively throughout the day, reaching higher values during sampling periods after midday, revealing a temporal pattern that parallels the intra‑day trends described in Figure 1c.

**Figure S8. Network-wide specialization degree** **across phenological blocks and flowering seasons.** Boxplots with accompanying violin plots depict the distribution of specialization degree (H2′) across 2‑h sampling intervals (6–8h, 8–10h, 10–12h, 12–14h, 14–16h, 16–18h), color‑coded along the orange–blue continuum to represent progression from early morning to late afternoon. Data are partitioned by flowering season (Season 1: 2021/2022; Season 2: 2022/2023) and subdivided into early, mid, and late phenological blocks (10 weeks each). Although seasonal structuring introduces some deviations, particularly during the early‑season, the dominant signal remains the within‑day variation: H2’ is highest in the early morning (6–8h), with a relative increase again toward dusk, revealing a temporal pattern broadly aligned with that described in Figure 1d.

**Figure S9. Weighted nestedness across phenological blocks and flowering seasons.** Boxplots with accompanying violin plots depict the distribution of weighted nestedness (ΔwNODF) across 2‑h sampling intervals (6–8h, 8–10h, 10–12h, 12–14h, 14–16h, 16–18h), color‑coded along the orange–blue continuum to represent progression from early morning to late afternoon. Data are partitioned by flowering season (Season 1: 2021/2022; Season 2: 2022/2023) and subdivided into early, mid, and late phenological blocks (10 weeks each). Although seasonal structuring introduces some deviations, particularly during the early‑season, the dominant signal remains the within‑day variation: ∆wNODF increases progressively throughout the day, reaching higher values during sampling periods after midday. This reveals a temporal pattern that parallels the intra‑day trends described in Figure 1e.

**Figure S10. Weighted modularity across phenological blocks and flowering seasons.** Boxplots with accompanying violin plots depict the distribution of weighted nestedness (ΔQw’) across 2‑h sampling intervals (6–8h, 8–10h, 10–12h, 12–14h, 14–16h, 16–18h), color‑coded along the orange–blue continuum to represent progression from early morning to late afternoon. Data are partitioned by flowering season (Season 1: 2021/2022; Season 2: 2022/2023) and subdivided into early, mid, and late phenological blocks (10 weeks each). Although seasonal structuring introduces some deviations, particularly during the early‑season, the dominant signal remains the within‑day variation: ∆Qw’ decreasing progressively throughout the day, reaching lower values toward dusk. This reveals a temporal pattern that parallels the intra‑day trends described in Figure 1f.

**Figure S11. Intra-seasonal phenological variation interaction β-diversity among daily sampling periods.** Panel (a) shows the distribution of interaction β-diversity (β^WN^) among all pairwise daily period comparisons, calculated separately for each phenological block (early-, mid-, late-season) after aggregating all interactions within each block for each plot and flowering season. Across blocks, β^WN^ values exhibit highly similar distributions, indicating that interaction β-diversity among daily periods follows comparable patterns at the beginning, middle, and end of the flowering season. Panels (b–d) show, for each phenological block (early-, mid-, late-season, respectively), the relationship between temporal distance between sampling periods (in hours) and interaction β-diversity (β^WN^). In all three blocks, interaction dissimilarity increases with increasing temporal distance, as indicated by the positive and significant Spearman correlations. Together, these results demonstrate that although species composition changes within the flowering season due to phenology, the temporal scaling of interaction β-diversity among daily periods remains consistent, and the higher β-diversity observed between more distant daily periods is independent of whether comparisons are made early, mid, or late in the flowering season.

**Figure S12. Plant–bee interaction β-diversity among each partial- and the full-day network and across phenological blocks and flowering seasons.** The bar plots represent the overall β-diversity of plant–bee interactions (i.e., βWN) among partial-day networks across phenological blocks (early, mid, late; each spanning 10 weeks) and flowering seasons (Season 1 and Season 2). Each bar corresponds to a partial-day network, indicating which sampling periods were combined to create the interaction matrix. Circles below the graphs are colored following the orange–blue continuum (from 6-8h – dark orange – to 16–18h – dark blue) and ordered according to the number of sampling periods that were merged to build the network. The numbers within each bar denote the number of sampling periods that were merged. Patterns across phenological blocks and flowering seasons follow the same trend described in Figure 4: interaction composition dissimilarities (βWN) between full- and partial-day networks generally decreased as additional 2-h sampling periods were included. Although some variation is evident, particularly in the early-season blocks, the representativeness of partial-day networks remains similar, with central 2-h periods (from 8-10h to 12-14h) being the most informative and consistently reducing β-diversity more strongly than other combinations.

**Table S1. List of bee taxa recorded in the study and their interaction patterns.** For each bee taxon, the table reports the type of floral resource collected (e.g., nectar, pollen, oil, resin), the main foraging behaviour (e.g., generalist, specialist, buzz-pollination, oil-collecting), the absolute frequency of interactions, and the relative frequency of interactions (expressed as the proportion of all recorded interactions in the dataset). In addition, the table details the distribution of interactions across the six 2-h daily sampling intervals, allowing the visualization of temporal variation in foraging activity throughout the day.

| **Bee species** | **Interaction frequency** | **% frequency** | **Foraging behaviour** | **Resource collected** | **Interaction timing** | | | | | |
| --- | --- | --- | --- | --- | --- | --- | --- | --- | --- | --- |
|  |  |  |  |  | 6-8h | 8-10h | 10-12h | 12-14h | 14-16h | 16-18h |
| *Acanthopus excellens* | 6 | 0.02 | - | Nectar/Pollen | 0 | 3 | 0 | 3 | 0 | 0 |
| *Agapostemum* sp1 | 3 | 0.01 | Buzz | Nectar/Pollen | 0 | 0 | 1 | 1 | 1 | 0 |
| *Ancyloscelis apiformis* | 21 | 0.09 | - | Nectar/Pollen | 1 | 8 | 6 | 5 | 1 | 0 |
| Anthidiini sp1 | 15 | 0.06 | - | Nectar/Pollen | 0 | 4 | 3 | 5 | 2 | 1 |
| *Apis mellifera* | 11858 | 48.31 | - | Nectar/Pollen | 1343 | 3389 | 4881 | 1450 | 546 | 249 |
| *Augochlora* sp1 | 21 | 0.09 | Buzz | Nectar/Pollen | 0 | 6 | 2 | 5 | 5 | 3 |
| *Augochloropsis* sp1 | 207 | 0.84 | Buzz | Nectar/Pollen | 14 | 46 | 51 | 35 | 41 | 20 |
| *Augochloropsis* sp2 | 5 | 0.02 | Buzz | Nectar/Pollen | 0 | 1 | 0 | 1 | 2 | 1 |
| *Augochloropsis* sp3 | 116 | 0.47 | Buzz | Nectar/Pollen | 4 | 24 | 41 | 18 | 19 | 10 |
| *Bombus morio* | 731 | 2.98 | Buzz | Nectar/Pollen | 111 | 179 | 113 | 130 | 109 | 89 |
| *Bombus pauloensis* | 52 | 0.21 | Buzz | Nectar/Pollen | 9 | 13 | 3 | 5 | 7 | 15 |
| *Centris* sp1 | 9 | 0.04 | Oil/Buzz | Nectar/Pollen/Oil | 1 | 1 | 0 | 2 | 3 | 2 |
| *Centris* sp2 | 154 | 0.63 | Oil/Buzz | Nectar/Pollen/Oil | 0 | 11 | 35 | 61 | 44 | 3 |
| *Centris* sp3 | 584 | 2.38 | Oil/Buzz | Nectar/Pollen/Oil | 102 | 144 | 115 | 96 | 83 | 44 |
| *Centris* sp4 | 23 | 0.09 | Oil/Buzz | Nectar/Pollen/Oil | 0 | 22 | 1 | 0 | 0 | 0 |
| *Centris* sp5 | 71 | 0.29 | Oil/Buzz | Nectar/Pollen/Oil | 6 | 4 | 6 | 11 | 26 | 18 |
| *Centris* sp6 | 29 | 0.12 | Oil/Buzz | Nectar/Pollen/Oil | 19 | 10 | 0 | 0 | 0 | 0 |
| *Centris* sp7 | 24 | 0.10 | Oil/Buzz | Nectar/Pollen/Oil | 5 | 7 | 2 | 1 | 5 | 4 |
| *Centris* sp8 | 63 | 0.26 | Oil/Buzz | Nectar/Pollen/Oil | 19 | 18 | 5 | 10 | 7 | 4 |
| *Ceratalictus* sp1 | 115 | 0.47 | Buzz | Nectar/Pollen | 9 | 29 | 27 | 19 | 17 | 14 |
| *Ceratina* sp1 | 4 | 0.02 | - | Nectar/Pollen | 0 | 0 | 2 | 1 | 0 | 1 |
| *Ceratina* sp2 | 1611 | 6.56 | - | Nectar/Pollen | 85 | 379 | 441 | 358 | 228 | 120 |
| *Dialictus* sp1 | 1 | 0.00 | - | Nectar/Pollen | 0 | 0 | 0 | 1 | 0 | 0 |
| Emphorini sp2 | 22 | 0.09 | - | Nectar/Pollen | 0 | 13 | 4 | 2 | 2 | 1 |
| Emphorini sp3 | 62 | 0.25 | - | Nectar/Pollen | 9 | 25 | 16 | 8 | 4 | 0 |
| Emphorini sp4 | 82 | 0.33 | - | Nectar/Pollen | 3 | 13 | 18 | 25 | 18 | 5 |
| *Epicharis* sp1 | 333 | 1.36 | Oil/Buzz | Nectar/Pollen/Oil | 142 | 51 | 17 | 23 | 33 | 67 |
| *Epicharis* sp2 | 145 | 0.59 | Oil/Buzz | Nectar/Pollen/Oil | 45 | 29 | 17 | 9 | 18 | 27 |
| *Epicharis* sp3 | 255 | 1.04 | Oil/Buzz | Nectar/Pollen/Oil | 118 | 65 | 27 | 17 | 15 | 13 |
| *Epicharis* sp4 | 16 | 0.07 | Oil/Buzz | Nectar/Pollen/Oil | 0 | 7 | 2 | 1 | 2 | 4 |
| Eucerini sp2 | 5 | 0.02 | - | Nectar/Pollen | 0 | 1 | 2 | 2 | 0 | 0 |
| *Eufriesea* sp1 | 16 | 0.07 | Buzz | Nectar/Pollen/Fragrance | 4 | 8 | 1 | 0 | 0 | 3 |
| *Euglossa* sp1 | 8 | 0.03 | Buzz | Nectar/Pollen/Fragrance | 0 | 1 | 4 | 2 | 0 | 1 |
| *Eulaema* *nigrita* | 169 | 0.69 | Buzz | Nectar/Pollen/Fragrance | 51 | 51 | 28 | 9 | 8 | 22 |
| *Exomalopsis* *analis* | 5 | 0.02 | Buzz | Nectar/Pollen | 1 | 0 | 3 | 0 | 1 | 0 |
| *Exomalopsis* *fulvofasciata* | 50 | 0.20 | Buzz | Nectar/Pollen | 3 | 12 | 7 | 9 | 11 | 8 |
| *Frieseomelitta* *varia* | 13 | 0.05 | - | Nectar/Pollen/Resin | 0 | 8 | 3 | 1 | 0 | 1 |
| *Mesoplia* sp1 | 112 | 0.46 | - | Nectar/Pollen | 2 | 4 | 27 | 23 | 26 | 30 |
| *Nannotrigona* sp1 | 20 | 0.08 | - | Nectar/Pollen | 0 | 4 | 0 | 13 | 1 | 2 |
| *Oxaea* *flavescens* | 43 | 0.18 | Buzz | Nectar/Pollen | 19 | 5 | 9 | 6 | 2 | 2 |
| *Paratrigona* sp1 | 2464 | 10.04 | - | Nectar/Pollen | 137 | 580 | 677 | 592 | 340 | 138 |
| *Paroxystoglossa* sp1 | 12 | 0.05 | Buzz | Nectar/Pollen | 0 | 2 | 5 | 0 | 4 | 1 |
| *Plebeia* sp1 | 32 | 0.13 | - | Nectar/Pollen | 7 | 11 | 3 | 2 | 7 | 2 |
| *Ptiloglossa* sp1 | 71 | 0.29 | Buzz | Nectar/Pollen | 71 | 0 | 0 | 0 | 0 | 0 |
| Tapinotaspidini sp1 | 26 | 0.11 | Oil | Nectar/Pollen/Oil | 0 | 3 | 4 | 14 | 5 | 0 |
| Tapinotaspidini sp2 | 116 | 0.47 | Oil | Nectar/Pollen/Oil | 24 | 23 | 35 | 18 | 10 | 6 |
| Tapinotaspidini sp3 | 247 | 1.01 | Oil | Nectar/Pollen/Oil | 14 | 65 | 94 | 57 | 14 | 3 |
| *Temnosoma* sp1 | 27 | 0.11 | - | Nectar/Pollen | 2 | 8 | 5 | 5 | 6 | 1 |
| *Tetragonisca* *angustula* | 648 | 2.64 | - | Nectar/Pollen/Resin | 8 | 56 | 263 | 278 | 42 | 1 |
| *Trigona* *spinipes* | 2725 | 11.10 | - | Nectar/Pollen/Resin | 173 | 425 | 668 | 541 | 508 | 410 |
| *Xylocopa* sp1 | 70 | 0.29 | Buzz | Nectar/Pollen | 31 | 32 | 1 | 0 | 4 | 2 |
| *Xylocopa* sp2 | 1029 | 4.19 | Buzz | Nectar/Pollen | 419 | 330 | 87 | 96 | 41 | 56 |

**Table S2. Mixed-model specifications used to test temporal variation in network structure and pairwise interaction turnover across sampling periods.** Response variables, predictor variables, random-effect structure, and error distributions used in the generalized linear mixed models (GLMMs) and linear mixed models (LMMs). Network structure metrics (pairwise interaction richness, connectance *C*, interaction evenness *IE*, specialization *H2’*, weighted nestedness *wNODF*, and weighted modularity *Qw’*) were modeled as a function of the 2-h sampling period, with season and plot identity included as crossed random effects. Pairwise interaction turnover (β^WN^) was modeled as a function of the comparison between sampling periods, using the same random-effect structure. Beta-regression models were fitted using a logit link, Poisson models used a log link, and Gaussian models used an identity link.

| **Response variable** | **Predictor** | **Random effects** | **Error distribution** | **Link function** |
| --- | --- | --- | --- | --- |
| Pairwise interaction richness | Sampling period (2-h) | Season + Plot | Poisson | Log |
| *C* (connectance) | Sampling period (2-h) | Season + Plot | Beta | Logit |
| *IE* (interaction evenness) | Sampling period (2-h) | Season + Plot | Beta | Logit |
| *H2’* (specialization degree) | Sampling period (2-h) | Season + Plot | Beta | Logit |
| *wNODF* (weighted nestedness) | Sampling period (2-h) | Season + Plot | Gaussian | Identity |
| *Qw’* (weighted modularity) | Sampling period (2-h) | Season + Plot | Beta | Logit |
| β^WN^ | Comparison between sampling periods | Season + Plot | Beta | Logit |

**Table S3**. **Full model selection table (AICc) for testing whether intraseasonal blocks (early-, mid-, and late-season) modify daily patterns of interaction β-diversity (β^WN^).** Models are generalized linear mixed models with beta error distribution, including plot identity as a random effect in all models. Fixed effects include daily period comparisons (DC), phenological structure (PS), flowering season (Season), and their interactions. Models were ranked by AICc using the dredge function in the ‘MuMIn’ package. In the table, “+” indicates that the variable or interaction term is included in the model, whereas “–” indicates that it is not included. The Akaike weight (Weight in the Table) represents the relative probability that a given model is the best-supported model among the candidate set, given the data and the set of models considered. Only two models received any substantial support (ΔAICc ≤ 4; above the dashed line), and none of them includes the interaction between daily period comparisons and phenological block, indicating that intra-seasonal phenological variation does not modify the daily pattern of interaction β-diversity.

| **Model** | **DC** | **PS** | **Season** | **DC×PS** | **DC×Season** | **PS×Season** | **DC×PS×Season** | **df** | **logLik** | **ΔAICc** | **Weight** |
| --- | --- | --- | --- | --- | --- | --- | --- | --- | --- | --- | --- |
| 40 | + | + | + | – | + | – | – | 22 | 729.953 | 0.00 | 0.88 |
| 8 | + | + | + | – | – | – | – | 20 | 725.871 | 3.99 | 0.12 |
| 56 | + | + | + | + | – | + | – | 50 | 751.758 | 16.71 | 0.00 |
| 24 | + | + | + | + | – | – | – | 48 | 747.586 | 20.63 | 0.00 |
| 48 | + | + | + | – | + | – | – | 36 | 733.494 | 22.64 | 0.00 |
| 16 | + | + | + | – | – | – | – | 34 | 729.398 | 26.53 | 0.00 |
| 64 | + | + | + | + | – | + | – | 64 | 755.637 | 40.45 | 0.00 |
| 32 | + | + | + | + | – | – | – | 62 | 751.481 | 44.21 | 0.00 |
| 6 | + | + | – | – | – | – | – | 19 | 695.774 | 62.10 | 0.00 |
| 128 | + | + | + | + | – | + | + | 92 | 776.165 | 65.31 | 0.00 |
| 22 | + | + | – | + | – | – | – | 47 | 717.252 | 79.09 | 0.00 |
| 39 | – | + | + | – | – | – | – | 8 | 649.753 | 131.51 | 0.00 |
| 7 | – | + | + | – | – | – | – | 6 | 647.436 | 132.09 | 0.00 |
| 5 | – | – | + | – | – | – | – | 5 | 622.078 | 180.78 | 0.00 |
| 4 | + | + | – | – | – | – | – | 18 | 506.200 | 439.17 | 0.00 |
| 2 | + | – | – | – | – | – | – | 17 | 496.045 | 457.41 | 0.00 |
| 12 | + | + | – | – | – | + | – | 32 | 511.286 | 458.48 | 0.00 |
| 3 | – | + | – | – | – | – | – | 4 | 445.701 | 531.51 | 0.00 |
| 1 | – | – | – | – | – | – | – | 3 | 437.163 | 546.57 | 0.00 |

| **Model step** | **Biological meaning** | **Fixed effects added** | **R² marginal** | **ΔR²** | **Cumulative R²** |
| --- | --- | --- | --- | --- | --- |
| M0 | Spatial baseline (i.e., plot as random effect) | — | 0.000 | — | 0.000 |
| M1 | Intraday temporal structure | Comparison among daily periods | 0.568 | **0.568** | 0.568 |
| M2 | Seasonal phenological structure | * Phenological period (i.e., early-, mid-, late-season) | 0.881 | **0.313** | 0.881 |
| M3 | Flowering season & interactions | * Flowering season (i.e., first and second season) | 0.901 | **0.020** | 0.901 |
| Conditional R² (full model) = 0.932; Marginal R² (full model) = 0.901. | | | | | |

**Table S4. Hierarchical variance partitioning of interaction β-diversity (β^WN^) across intraday and seasonal temporal scales.** We fitted sequential generalized linear mixed models with beta error distribution, including plot identity as a random effect. Marginal R² indicates the variance explained by fixed effects, while conditional R² includes both fixed and random effects. ΔR² represents the incremental increase in explained variance after adding biologically meaningful blocks of predictors and interaction terms: intraday temporal structure, seasonal phenological structure (i.e., early-, mid-, and late-season), and flowering season (i.e., first and second flowering season; full model). Random effects alone (M0) explained 0% of the variance. Adding intraday temporal structure (Comparison; M1) explained 56.8% of variance (ΔR² = 0.568). Incorporating seasonal phenological structure (Phenological period; M2) increased explained variance to 88.1% (ΔR² = 0.313). Including flowering season and interactions (M3) resulted in only a minor increase to 90.1% (ΔR² = 0.020). These results indicate that the majority of variation in interaction β-diversity is attributable to intraday temporal structure, while seasonal phenological and flowering season effects contribute much less. The full model’s conditional R² = 0.932 confirms that random plot-to-plot variation adds relatively little additional explanatory power.

**References**

Ballarin, C. S. Amorim, F. W. Arroyo-Correa, B. & Jordano, P. (2026). Floral resource diversity drives spatiotemporal variation in plant–pollinator network structure. *Oikos*, DOI: 10.1002/oik.11730.

Nakagawa, S., & Schielzeth, H. (2013). A general and simple method for obtaining R2 from generalized linear mixed‐effects models. Methods in ecology and evolution, 4(2), 133-142.
